# PLAU directs conversion of fibroblasts to inflammatory cancer-associated fibroblasts, promoting esophageal squamous cell carcinoma progression via uPAR/Akt/NF-κB/IL8 pathway

**DOI:** 10.1038/s41420-021-00410-6

**Published:** 2021-02-11

**Authors:** Lingling Fang, Yun Che, Chaoqi Zhang, Jianbing Huang, Yuanyuan Lei, Zhiliang Lu, Nan Sun, Jie He

**Affiliations:** Department of Thoracic Surgery, National Cancer Center/National Clinical Research Center for Cancer/Cancer Hospital, Chinese Academy of Medical Sciences and Peking Union Medical College, Beijing, China

**Keywords:** Cancer microenvironment, Prognostic markers

## Abstract

Cancer-associated fibroblasts (CAFs) plays an important role in the tumor microenvironment. The heterogeneity of CAFs affects the effect of CAFs on promoting or inhibiting tumors, which can be regulated by other cells in the tumor microenvironment through paracrine methods. The urokinase-type plasminogen activator (PLAU) system mediates cell proliferation, migration, adhesion, and other functions through the proteolytic system, intracellular signal transduction, and chemokine activation. PLAU promotes tumor progression in many tumors. We explored the function of PLAU in ESCC and the influence of PLAU secreted by tumor cells on the heterogeneity of CAFs. We found that PLAU is highly expressed in ESCC, which is related to poor prognosis and can be used as a prognostic marker for ESCC. Through loss-of function and gain-of function experiments, we found that PLAU promoted ESCC proliferation and clone formation via MAPK pathway, and promotes migration by upregulating Slug and MMP9, which can be reversed by the MEK 1/2 inhibitor U0126. At the same time, through sequencing, cytokine detection, and RT-qPCR verification, we found that tumor cells secreted PLAU promoted the conversion of fibroblasts to inflammatory CAFs, which upregulated expression and secretion of IL8 via the uPAR/Akt/NF-κB pathway. The IL8 secreted by CAFs in turn promotes the high expression of PLAU in tumor cells and further promoted the progression of ESCC. In summary, PLAU was not only a prognostic marker of ESCC, which promoted tumor cell proliferation and migration, but also promoted the formation of inflammatory CAFs by the PLAU secreted by tumor cells.

## Introduction

Esophageal cancer is one of the most common cancers in the world^1^. In 2015, there were an estimated 478,000 new cases and 375,000 deaths in China^2^. Esophageal cancer is highly malignant, and the prognosis is poor. The five-year survival rate is 15–25%^3^. Esophageal squamous cell carcinoma (ESCC) is the main pathological type of esophageal cancer. Surgery combined with radiotherapy and chemotherapy is the main treatment for ESCC. However, drug resistance, recurrence, and metastasis are prone to appear after treatment, and the overall quality of life and prognosis of patients are poor.

Cancer-associated fibroblasts (CAFs) are an important component of the tumor microenvironment^4^. They mainly act by secreting cytokines, changing the extracellular matrix, and interacting with tumor cells and other cells^4–6^. Many studies have confirmed that CAFs play an important role in the occurrence, development, drug resistance, and metastasis of ESCC by secreting various cytokines, including HGF, IL-6, CXCL1, and PAI-1^7–10^. There is enough evidence to prove that CAFs play an important role in tumor progression and may represent new targets for tumor therapy^11^. Targeting CAFs not only inhibits the growth of tumor “seeds” but also modifies the “soil” to generate a microenvironment that inhibits tumor proliferation and metastasis^4^. Although it is generally believed that CAFs mainly play a role in promoting tumors, some studies have found that the targeted elimination of CAFs in pancreatic cancer leads to tumor deterioration and progression. This finding demonstrates that the greatest challenge of targeting CAFs to treat tumors is the heterogeneity of CAFs^11–15^. In addition, as prognostic markers, CAFs have also demonstrated the opposite results in many studies^11,13^. CAFs can be divided into different subgroups, such as tumor-promoting CAFs (pCAFs), tumor-inhibiting CAFs (rCAFs), neutral CAFs (nCAFs), inflammatory CAFs (iCAFs), and myofibroblasts (myCAFs)^16–18^.

The urokinase-type plasminogen activator (PLAU) system includes PLAU, urokinase plasminogen activator receptor (uPAR) and its inhibitor PAI-1. This system mediates cell proliferation, migration, adhesion, and other functions through the proteolytic system, intracellular signal transduction, and chemokine activation. This system is related to a variety of pathological and physiological processes and plays an important role in tumor occurrence and development^19^. PLAU is overexpressed in many tumors and plays an important role in tumor development and metastasis^20–22^. PLAU is a biomarker and prognostic indicator of breast cancer, which promotes trastuzumab resistance^23^. In addition, exosomes overexpressing of mir-23b and miR-320b in epithelial cells in breast cancer upregulate the expression of PLAU^24^. Our previous studies have confirmed that CAF-derived PAI-1 promotes ESCC cell proliferation and clone formation, and inhibits ESCC cell apoptosis caused by cisplatin. In vivo, PAI-1 can promote ESCC cell subcutaneous tumor formation in nude mice and cisplatin resistance. PAI-1 expression in the drug-resistant CAFs group was significantly increased and associated with poor progression-free survival (PFS)^8^. This study aims to explore the function of PLAU in ESCC tumor cells and the influence of PLAU secreted by tumor cells on CAF heterogeneity in the microenvironment.

## Materials and methods

### Patients and tumor samples

A paraffin-embedded ESCC microarray contained 55 ESCC tissue with 50 adjacent tissues was purchased from Outdo Biotech (HEsoS105Su01) for immunohistochemistry studies. Ten fresh ESCC tumor and paratumor tissues were obtained from our hospital for isolation of CAFs in 2019. All patients signed informed consent. Our study was approved by the Committee for the Ethics Review of Research Involving Human Subjects of the Cancer Hospital of the Chinese Academy of Medical Sciences.

### Immunohistochemistry (IHC) and scoring

In brief, IHC was performed as reported in a previous study. Anti-PLAU antibody (diluted 1:100, ab169754, Abcam) was used. The final immunoreactivity score (IRS) was the product of staining intensity and percentage of positive cells.

### Cell culture and stable cell construction

As previously described, the ESCC cell lines KYSE-30, KYSE-70, KYSE-140, KYSE-150, KYSE-180, KYSE-450, and KYSE-510 were cultured in RPMI 1640 medium (HyClone)^25^, and Het-1a esophageal epithelial cells were cultured in BEGM BulletKit medium (Lonza/Clonetics). MRC-5 (Medical Research Council cell strain-5, KG508, Keygene) was cultured in MEM (minimum Eagle’s medium) with 10% fetal bovine serum, 1% nonessential amino acid and 1 mM sodium pyruvate. Full-length *PLAU* cDNA was synthesized by SyngenTech (China) and ligated into a pLV-CMV-Puro vector. Two short hairpin RNA (shRNA) oligonucleotides (5′-GCATGACTTTGACTGGAATTG-3′ and 5′-GCAGTAGAGTCATCTCCATCA-3′) were inserted into pLV-Puro (SyngenTech, China). The negative control (NC) sequence was 5′-AAACGTGACACGTTCGGAGAA-3′. As previously described, we constructed ESCC cells with knockdown or overexpression of *PLAU* and controls. PLAU expression in infected cells was confirmed by RT-qPCR and western blot 96 h after infection.

### Isolation CAFs and normal fibroblasts (NFs)

Homogeneous CAFs or NFs were isolated from fresh tumor or paratumor tissues and were identified using the cellular immunofluorescence marker αSMA as noted in our previous study^8^. The patients’ characteristics was in Supplementary Table 2. All CAFs and NFs used in the study were grown for no more than ten passages.

### Collection of conditional medium (CM)

In brief, the CM was collected after shPLAU-1, shPLAU-2, -vec KYSE-30, and KYSE-450 cells and *PLAU*, vector KYSE-180 and KYSE-450 cells were cultured in serum-free medium for 24 h. The CM of CAFs was collected as follows. After exposure to various treatments (stimulated by drugs or CM of tumor cells, or cocultured with tumor cells), the medium was replaced with the serum-free medium, and cells were incubated for 24 h. CM was centrifuged at 1000×*g* for 5 min for further experiments. For western blot, CM was concentrated 40-fold using a Centricon Centrifugal filter (Millipore, USA). For cell stimulation, CM was sterile filtered and diluted once with the medium.

### Drugs

Briefly, 10 μM U0126 (catalog no. S1102, Selleck) was added to the cell culture for 1 h to inhibit MEK1/2 in vitro. CAFs were treated with 2 ng/ml recombinant PLAU (catalog no. ab167764, Abcam) for 24 h. Briefly, 10 μM IPR-803 (catalog no. HY-111192, Selleck) was added 1 h prior to inhibiting PLAU-uPAR and before any other treatments. Recombinant IL8 (catalog no. 500-P28, Peprotech) was used to at final concentrations of 5 ng/ml unless specified. Times of treatments were 24 h unless specified.

### Coculture system

For the coculture system, WT KYSE-30 and KYSE-450 cells were seeded in a 24-well plate, and CAFs with recombinant PLAU and IPR-803 treatments were placed in the upper chamber with Matrigel with a 0.4-μm pore size (catalog no. 3412, Corning). Cells were cocultured for 48 h. Then, KYSE-30 and KYSE-450 cell proliferation was detected using the CCK8 assay. In another experiment, CAFs treated with IPR-803 or PBS were seeded in a 24-well plate, and KYSE-450 cells overexpressing *PLAU* were seeded in the upper chamber. After coculture for 48 h, proteins were extracted from CAFs. Alternatively, the serum-free medium was replaced, and cells were incubated for 24 h. Then, CM of CAFs was collected for further experiments.

### RNA extraction and quantitative real time PCR (RT-qPCR)

RNA extraction and RT-qPCR were performed as described in a previous study^25^. The 2^−ΔΔCt^ method was used to quantify the relative RNA expression level, and β-Actin served as an endogenous reference. All primers and oligonucleotides used in this study are listed in Supplementary Table S3.

### Western blotting

Western blotting was performed as previously described^25^. The primary antibodies used in the study are listed in Supplementary Table S4.

### Luminex liquid suspension chip detection

CM of CAFs with or without PLAU treatment were used in these studies. Each sample was assessed in duplicate. Luminex liquid suspension chip detection was performed by Wayen Biotechnologies (Shanghai, China).

### Enzyme-linked immunosorbent assay (ELISA)

CM of CAFs subject to various treatments (stimulated by drugs or CM of tumor cells, or cocultured with tumor cells) was collected for measurement of IL8 protein (without dilution) levels using the Human IL8 ELISA kit (ELH-IL8-1, RayBio). In addition, PLAU protein was measured in CM of knockdown and overexpressing *PLAU* KYSE-30, KYSE-450, and KYSE-180 cells and CM of wild type (WT) KYSE-30 and KYSE-450 cells using the PLAU ELISA kit (ELH-uPA-1, RayBio). Each sample was assessed in duplicate wells. Concentrations were calculated according to the manufacturer’s instructions.

### RNA sequencing

Here, sh*PLAU*-1, -vec KYSE30 and KYSE450 cells were subject to mRNA sequencing (mRNA-seq) using Illumina HiSeq 4000. NFs and CAFs treated with PBS or recombinant PLAU were also assessed. mRNA-seq was performed by Boao (China).

### Cell proliferation and colony formation

CCK8 assays (KeyGEN, China) were performed followed by the manufacturer’s instructions. In brief, cells (KYSE-30: 2000 cells/well; KYSE-180 and KYSE-450: 2500 cells/well) were seeded into 96-well plates, and the OD values were measured at 0, 24, 48, and 72 h. OD values of KYSE-450 cells cocultured with CAFs were measured at 48 h. For the colony formation assay, 200 cells were seeded into 6-well plates and cultured for 10–14 days. The colonies were fixed and stained with 1% crystal violet.

### Boyden chamber transwell assays

In brief, we used 24-well Boyden chambers (Corning, USA) for migration assays as previously described^25^. Transfected cells (KYSE30,1 × 10^4^ or KYSE450, 5 × 10^4^) were plated into the upper chamber and cultured in serum-free RPMI 1640 medium with or without treatment (cells overexpressing *PLAU* with U0126 added in the upper chamber). WT KYSE-30 and KYSE-450 cells were treated with CM of CAFs with or without recombinant PLAU in the presence or absence IPR-803. We counted the numbers of cells that migrated in five different areas at 100-fold magnification.

### Tumor xenograft experiment

All the animal experiments were approved by the Institutional Animal Care and Use Committee of the Cancer Hospital, Chinese Academy of Medical Sciences, and Peking Union Medical College. As previously described^26^, 1 × 10^6^ sh*PLAU*-1, -vec KYSE-30 cells, and KYSE-180 cells overexpressing *PLAU* and vector KYSE-180 cells were subcutaneously injected into the flanks of mice (female 6-week-old to 8-week-old nude mice, *n* = 7 per group) to establish tumor xenografts. In another experiment, MRC-5 was stimulated with 20 ng/ml TGFβ1 for 4–5 days before further animal experiment. Briefly, 1 × 10^6^ WT KYSE-450 cells alone or 5 × 10^5^ WT KYSE-450 cells mixed with 5 × 10^5^ MRC-5 (or recombinant uPA pretreated MRC-5) were resuspended in 0.2 ml of PBS and then subcutaneously injected into the flanks of mice to establish tumor xenograft (female 6-week-old to 8-week-old nude mice, *n* = 7 per group). Subgroups of mice were administered IPR-803 (50 mg/kg) via the intragastric route once every three days when the tumor reached 5 mm in diameter. The tumor volume was calculated by the formula: *V* = (*L* × *W*^2^)/2. Four weeks later, all mice were sacrificed, and the tumor was removed and weighed.

### Lung colonization assay

In brief, 1 × 10^6^ sh*PLAU*-1, -vec KYSE-30, and overexpressing *PLAU* and vector KYSE-180 cells were injected into female NOD-SCID mice (4-week-old to 6-week-old, six per group) through the tail vein. The mice were sacrificed 7 weeks later. The lungs were excised and fixed with 4% polysorbate and subsequently embedded in paraffin for hematoxylin and eosin (H&E) staining. The number of lung surface metastatic nodes was calculated by gross and microscopic examination.

### Statistical analysis

Prism GraphPad version 6.0, SPSS, GSEA, R scripting, and Cistrome network were used. Correlations between IL8 and *PLAU* expression were analyzed using Pearson’s correlation coefficient. A chi square test was performed to determine the relationship between clinicopathological variables and PLAU expression. Overall survival (OS) curves were analyzed using the Kaplan–Meier method and log-rank tests. Significant differences between different groups were analyzed using two-tailed *t*-test or nonparametric Wilcoxon Sign-rank test. Data were presented as the mean standard deviation (SD). Differences were considered significant at *P* < 0.05, ****P < 0.0001, ***P < 0.001, **P < 0.01, and *P < 0.05 (ns, not significant).

## Results

### High *PLAU* expression could indicate a poor prognosis in ESCC tissue

**Fig. 1:**
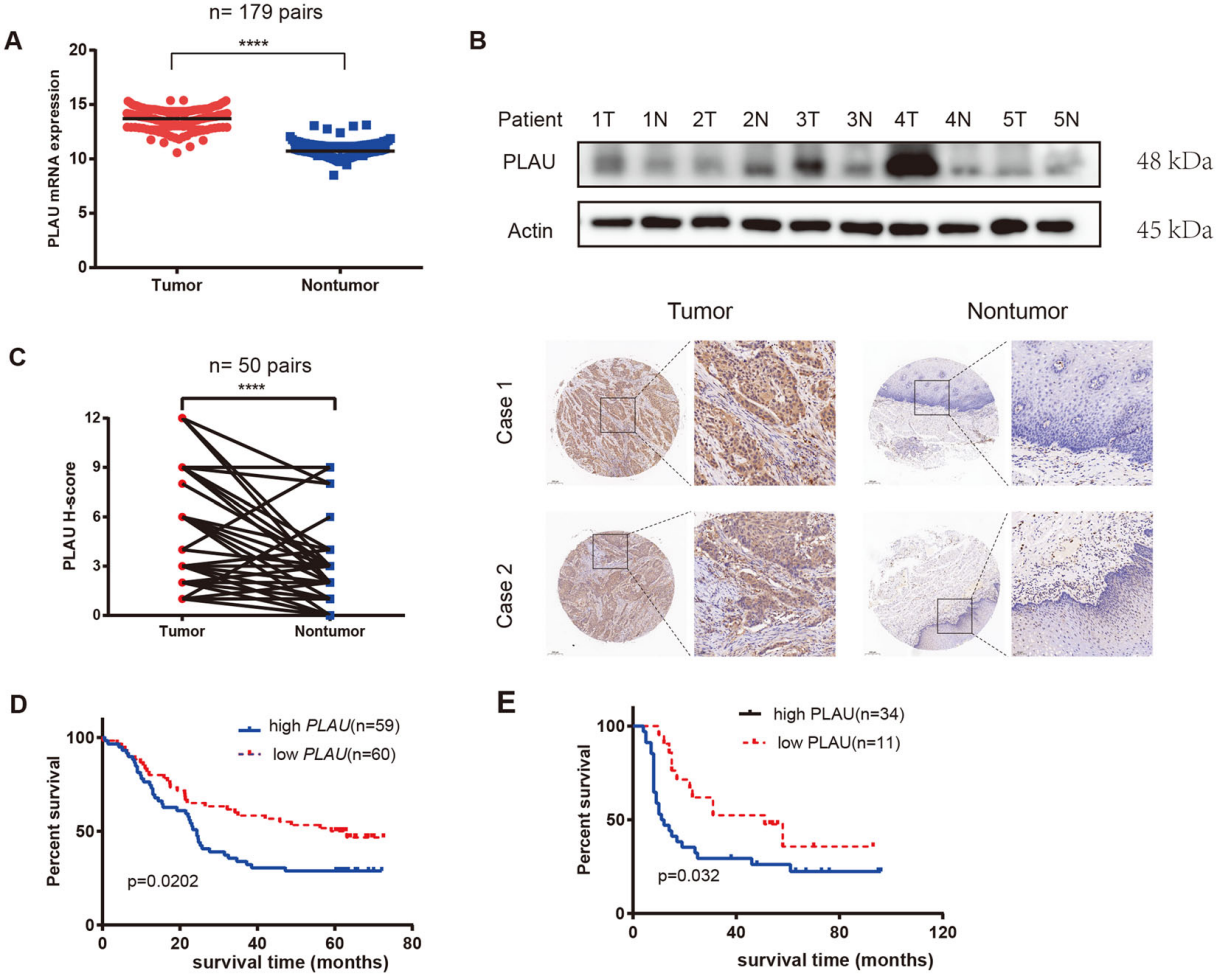
High PLAU expression indicates a poor prognosis in ESCC tissue. **A**, **B** PLAU was more highly expressed in cancer compared with para-cancer tissues at the RNA (**A**, GSE53625) and protein (**B**, **C**) levels, **B** western blot analysis of tumor and adjacent nontumor tissues from five patients, and **C** immunohistochemistry (IHC) scores (left) and representative images (right) of PLAU staining in ESCC tumor tissues and nontumor tissues (original magnification: 50×, 200×). **D**, **E** Kaplan–Meier survival analysis of overall survival based on high (*n* = 89) and low (*n* = 90) PLAU expression in GSE53625 (D) and high (*n* = 34) and low (*n* = 11) PLAU expression by IHC (**E**). *P < 0.05, **P < 0.01, ***P < 0.001, ****P < 0.0001 (Student’s *t*-test or).

By analyzing GSE53625 data, we found that *PLAU* RNA expression was increased in cancer tissue compared with adjacent tissue (Fig. 1A). Furthermore, at the protein level, PLAU upregulation was evident only in three out of five derived tissue patients tested (Fig. 1B). PLAU immunohistochemical staining was performed on the ESCC tissue microarray, including 55 tumor and 50 adjacent cancer tissue samples, and the result were consistent with that noted at the RNA level (Fig. 1C). In addition, high PLAU expression predicted poor prognosis at both the RNA and protein expression levels (Fig. 1D, E). All these findings indicate that PLAU can serve as a prognostic marker of ESCC.

### *PLAU* was critical for ESCC cell proliferation and tumor growth in vitro and in vivo

**Fig. 2:**
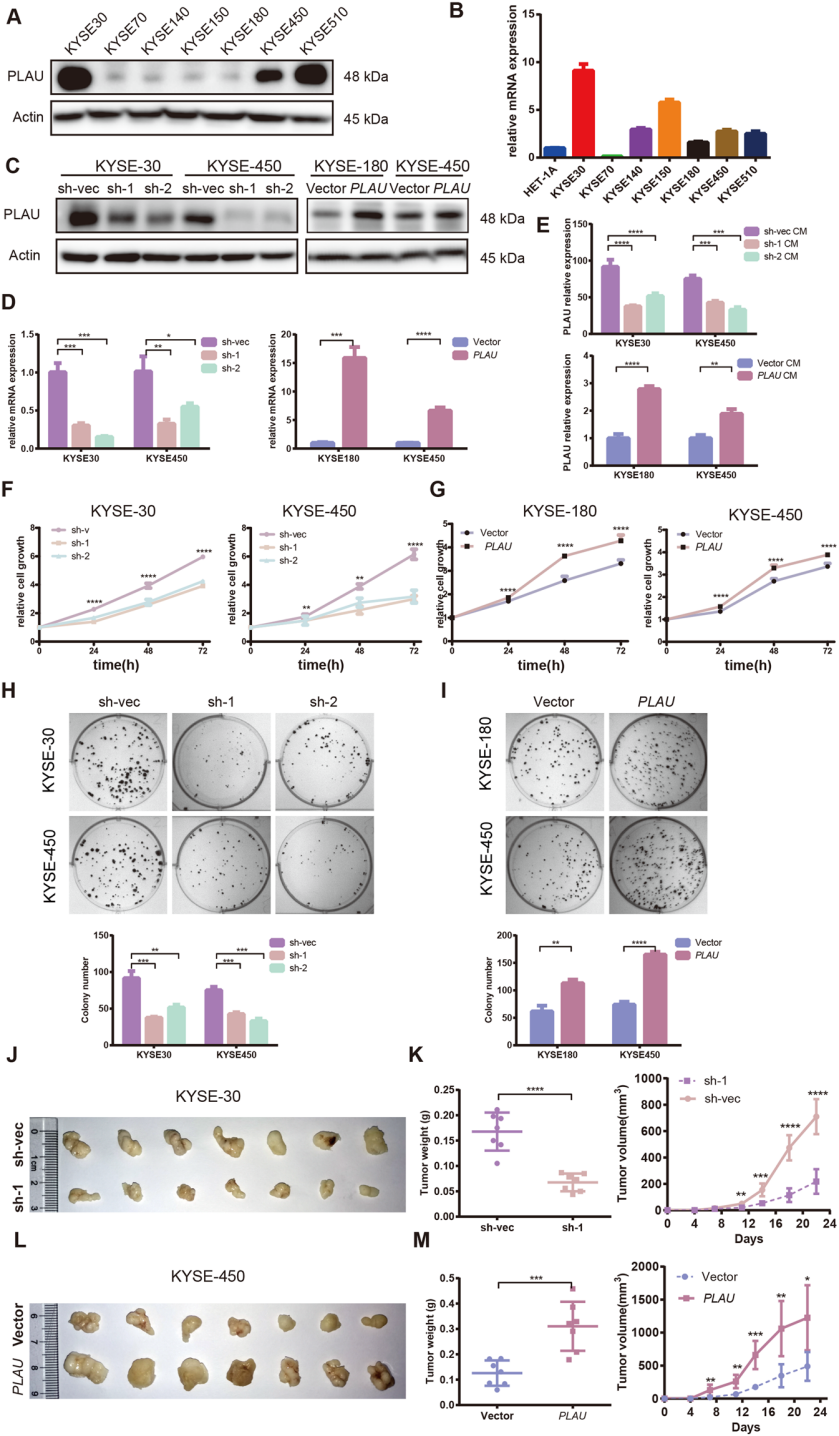
PLAU was critical for ESCC cell proliferation and tumor growth. **A**, **B** Expression of PLAU in seven ESCC cell lines at the protein and RNA level (relative to HET-1A). **C**–**E** *PLAU* knockdown or overexpression in ESCC cells was verified by western blot (**C**), RT-qPCR (**D**), and ELISA (E). **F**, **G** Rate of cell growth of sh*PLAU*-1, sh*PLAU*-2 and sh-vec KYSE-30 and KYSE-450 cells and *PLAU* overexpressing and vector control cells were measured by CCK8 assays. **H**, **I** Colony formation of *PLAU* knockdown and overexpression KYSE-30, KYSE-450, and KYSE-180 cells. Colony numbers were compared between the groups. **J**–**M** Photos, weights, and growth curves of tumors in nude mice subcutaneously inoculated with sh*PLAU*-1 and sh-vec KYSE-30 cells (**J**, **K**) or *PLAU* overexpressing or control cells (**L**, **M**) (female 6-week-old to 8-week-old nude mice, *n* = 7 per group). Three biological replicates were performed for in vitro assays. Data in bar charts are presented as the mean ± SD. *P < 0.05, **P < 0.01, ***P < 0.001, ****P < 0.0001 (Student’s *t*-test).

Previous studies have shown that *PLAU* can promote tumor proliferation in many tumors, such as breast cancer, and bladder cancer^22,27^. To explore *PLAU* function in ESCC, we detected PLAU expression levels in seven ESCC cell lines (Fig. 2A, B). Then, we constructed stable *PLAU* knockdown and overexpression ESCC cells and verified PLAU expression using WB and RT-qPCR (Fig. 2C, D). Given that PLAU is a secreted protein, we simultaneously assessed the supernatant of *PLAU* knockdown and overexpressing cells, and the results were consistent with that noted for total protein expression (Fig. 2E). There was no correlation between PLAU mRNA and protein expression in a number of cell lines. Using CCK8 experiments, we found that the proliferation of knockdown *PLAU* cells was reduced compared with the control, whereas the proliferation of cells overexpressing *PLAU* was enhanced (Fig. 2F, G). In addition, cells with *PLAU* knockdown and overexpression were subject to clone formation assays, and *PLAU* promoted the ability of ESCC cells to form clones (Fig. 2H, I). In vivo, the knockdown *PLAU* group exhibited reduced tumor growth and weight than the control group (Fig. 2J, K), while the *PLAU* overexpression group exhibited increased tumor growth and weight compared with the control group (Fig. 2L, M). These results show that *PLAU* promotes the growth of ESCC.

### *PLAU* promoted ESCC migration in vitro and in vivo

**Fig. 3:**
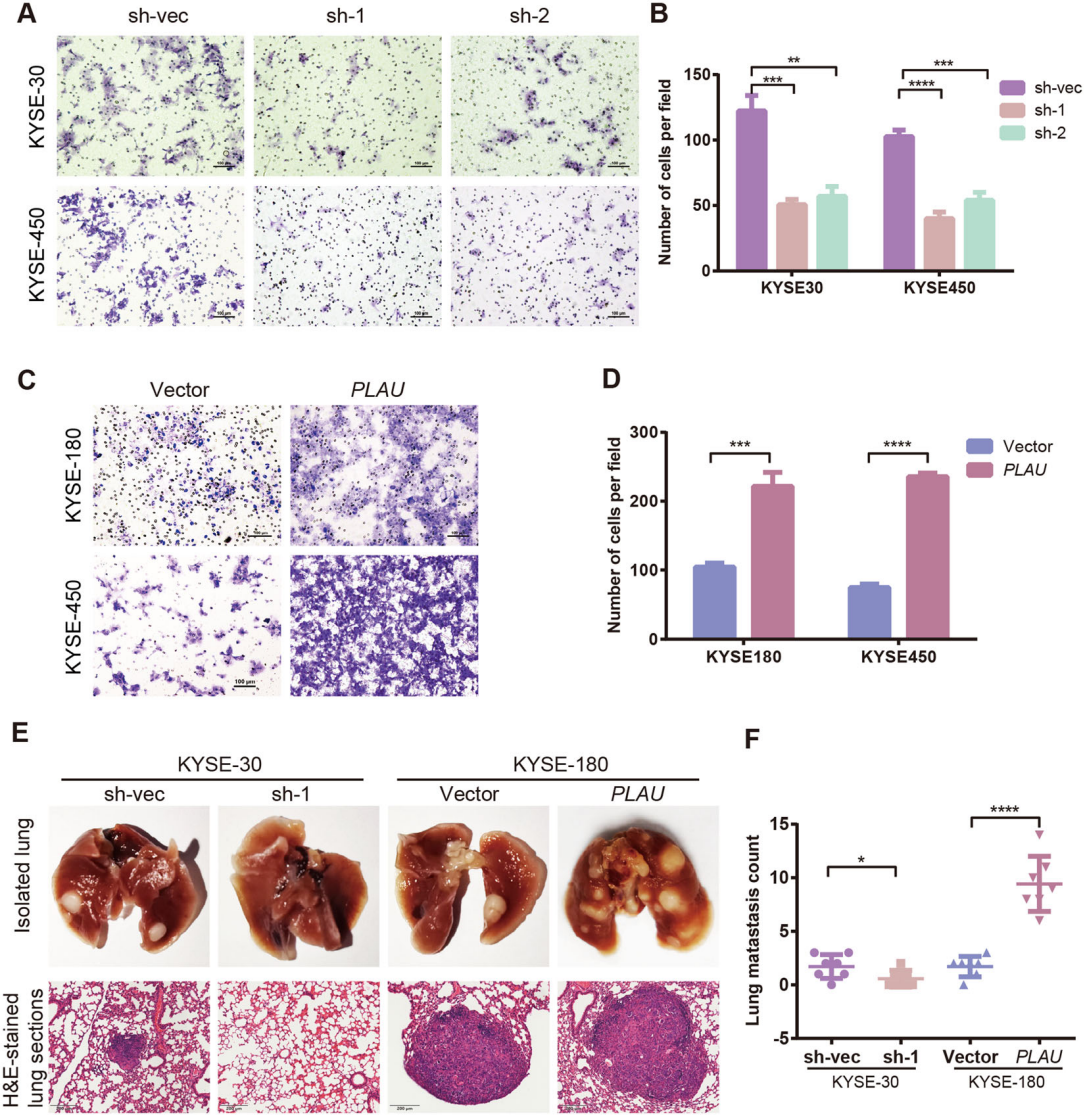
PLAU promoted ESCC cell migration in vitro and in vivo. **A**–**D** The migrating ability of sh*PLAU*-1, sh*PLAU*-2, sh-vec KYSE-30 and KYSE-450 cells (**A**) and *PLAU* overexpressing KYSE-180 and KYSE-450 cells (**C**) was detected using transwell chamber assays. The numbers of migrating cells were compared between the groups (**B**, **D**). **E**, **F** Representative images of lung tissues isolated from mice injected with 1 × 10^6^ sh*PLAU*-1 or sh-vec KYSE-30 cells, and *PLAU* overexpressing or vector KYSE-180 cells via the tail vein. Hematoxylin (the top panel) and eosin-stained images (200×, scale bars, 200 µm) of such tissues, and quantification of lung metastasis (female 4-week-old to 6-week-old NOID/SCID mice, *n* = 7 per group). Three biological replicates were performed for in vitro assays. Data in bar charts are presented as the mean ± SD. *P < 0.05, **P < 0.01, ***P < 0.001, ****P < 0.0001 (Student’s *t*-test).

*PLAU* promotes migration in a variety of tumors^27–29^. Using Boyden chamber transwell assays, we found that the migration ability of cells with *PLAU* knockdown was decreased compared with the control (Fig. 3A, B), whereas cells overexpressing *PLAU* exhibit the opposite results (Fig. 3C, D). In addition, in vivo experiments showed that the number of pulmonary nodules in the knockdown *PLAU* group was less than that in the control group, whereas the number of pulmonary nodules in the PLAU overexpression group increased (Fig. 3E, F).

### *PLAU* promoted ESCC cell proliferation and migration via activation of the MAPK/MEK/Erk/Slug/MMP9 pathway

**Fig. 4:**
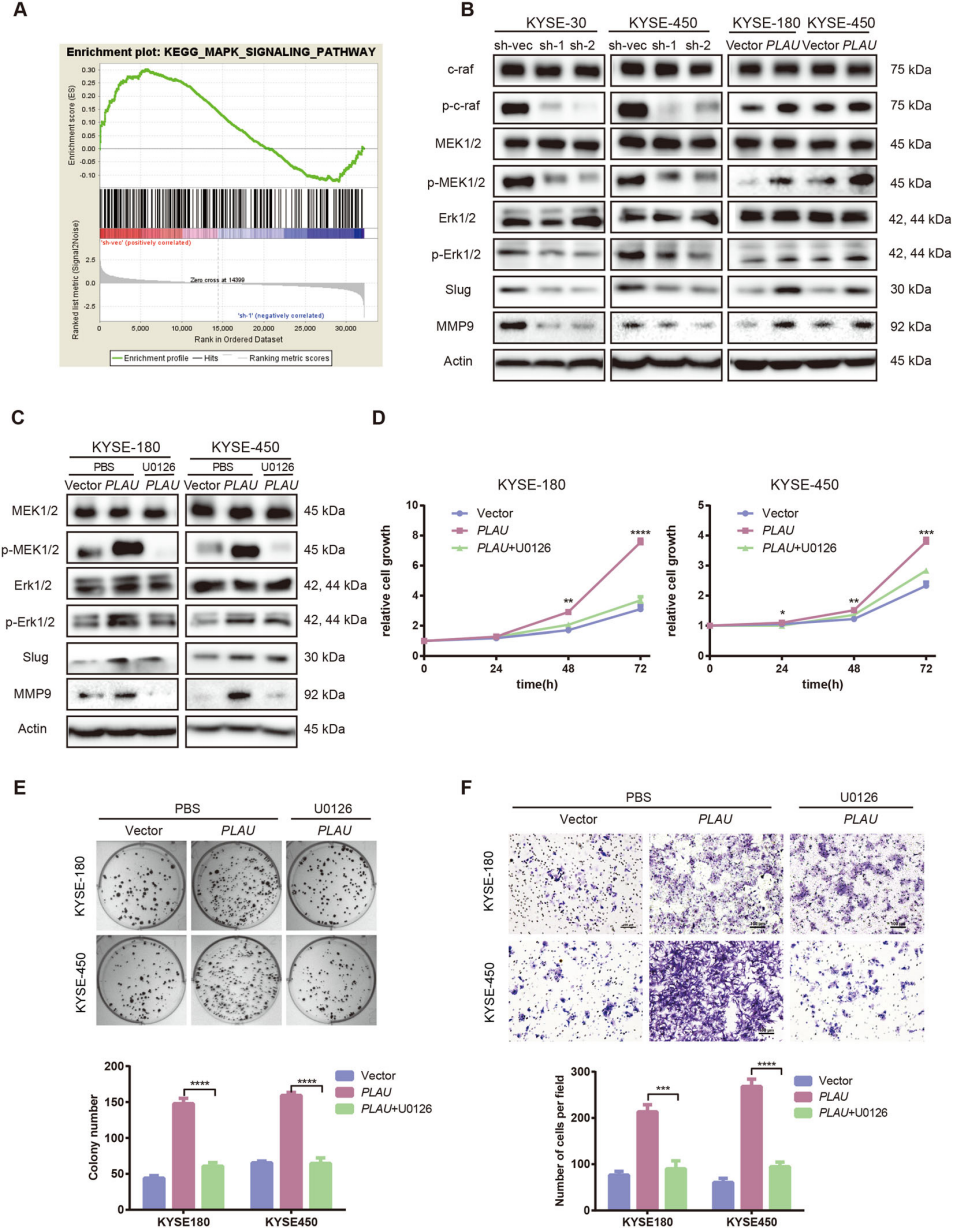
PLAU promoted ESCC cell proliferation and migration via activation of the MAPK/MEK/Erk/Slug/MMP9 pathway. **A** MAPK signaling pathways enriched by sh-vec using GSEA. **B**, **C** Total and phosphorylated c-raf, MEK1/2, Erk1/2, Slug, and MMP9 expression levels in sh*PLAU*-1, sh*PLAU*-2, and sh-vec KYSE-30 and KYSE-450 cells and overexpressing LAMC1 or vector KYSE-180 and KYSE-450 with (**B**) or without (**C**) U0126 treatment, a highly selective inhibitor of MEK1/2. **D**–**F** Proliferation, colony formation and migration of KYSE-180 and KYSE-450 cells overexpressing *PLAU* and vector with or without U0126 treatment. The number of colonies or migrating cells were compared between the groups (the bottom panel). Three biological replicates were performed for in vitro assays. Data in bar charts are presented as the mean ± SD. *P < 0.05, **P < 0.01, ***P < 0.001, ****P < 0.0001 (Student’s *t*-test).

To further explore the molecular mechanism by which *PLAU* promotes ESCC proliferation and migration, we performed RNA sequencing of sh*PLAU*-1 and sh-vec KYSE-30 and KYSE-450 cells. Based on enrichment analysis, we hypothesized that the MAPK signaling pathway may be responsible for *PLAU*-mediated ESCC progression (Fig. 4A). Then, we verified the role of the classic MAPK signaling pathway. Western blot was used to detect the total and phosphorylated protein levels of c-raf, MEK, and Erk1/2 in knockdown and overexpressing *PLAU* ESCC cells, respectively. The results revealed reduced phosphorylated c-raf, MEK, and Erk1/2 levels in knockdown *PLAU* cells compared with control, whereas the expression of these phosphorylated proteins was increased in cells overexpressing *PLAU* (Fig. 4B). Slug and Snail are common transcription factors that regulate invasion and metastasis in the downstream MAPK/MEK/Erk pathway. We further detected Slug and Snail expression levels in *PLAU* knockdown and overexpression cells. We found that PLAU upregulated Slug expression (Fig. 4B). MMPs is a common protein downstream of Slug that regulates migration. We found that MMP9 expression in *PLAU* knockdown cells was reduced compared with sh-vec cells, whereas MMP9 expression in cells overexpressing *PLAU* was increased (Fig. 4B). In addition, the highly selective MEK1/2 inhibitor U0126 reduce the high levels of MEK1/2 and Erk1/2 phosphorylation and Slug and MMP9 expression in cells overexpressing *PLAU* (Fig. 4C). In addition, U0126 also reversed *PLAU*-mediated promotion of ESCC cell proliferation, colony formation and migration (Fig. 4D–F).

### CAFs stimulated by PLAU promoted ESCC proliferation and migration

*PLAU* in tumor cells further promotes ESCC cell proliferation and invasion by activating the classic MAPK pathway. Our previous studies demonstrate that the expression of PAI-1 and uPAR, both of which belong to the PLAU system, are significantly increased in CAFs and tumor cells, respectively, and promote ESCC cell proliferation and drug resistance^8^.

**Fig. 5:**
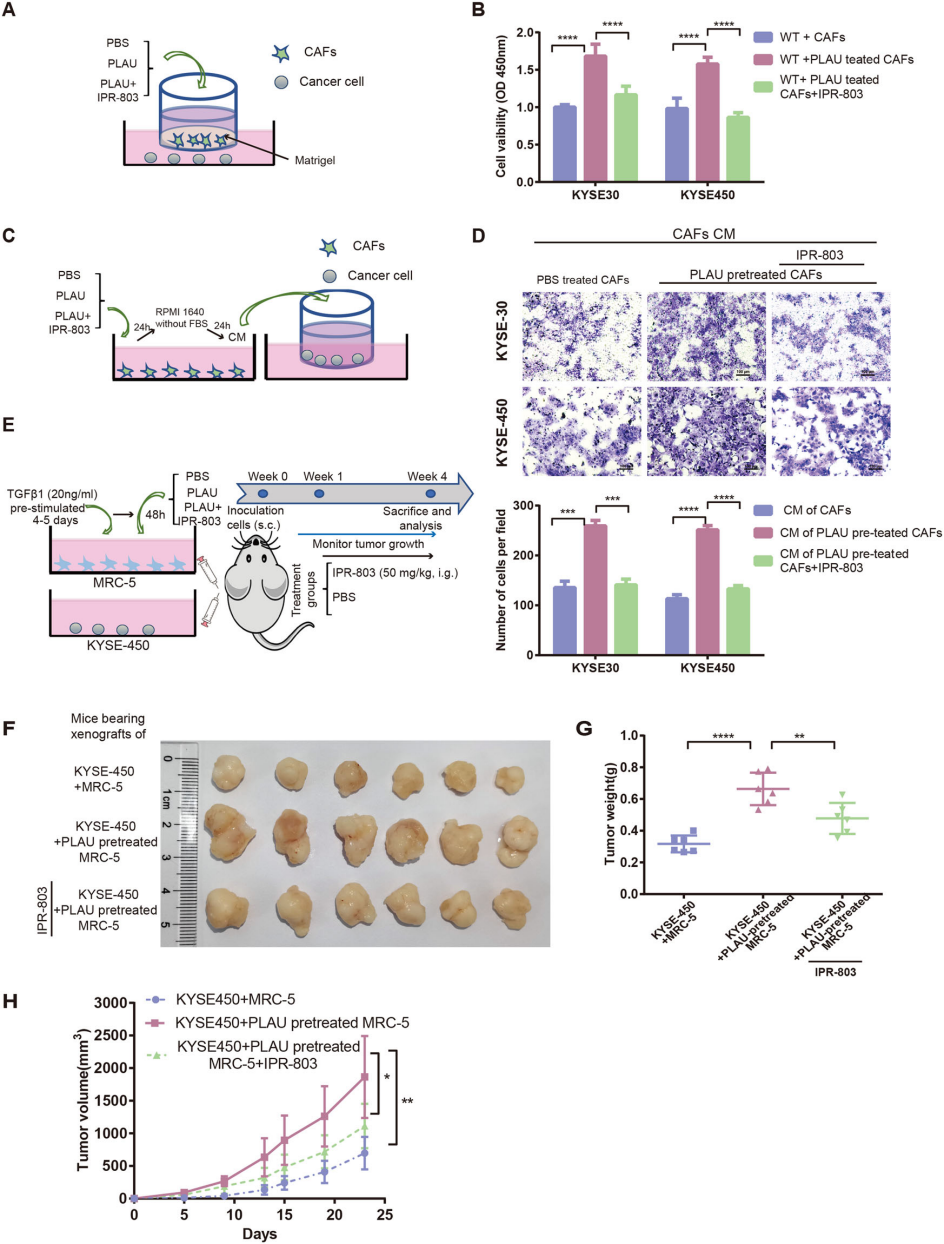
CAFs stimulated by PLAU promoted ESCC proliferation and migration. **A**, **B** Experimental scheme and growth of WT KYSE-30 and KYSE-450 cells cocultured with CAFs with or without recombinant PLAU in the presence or absence of the PLAU-uPAR inhibitor IPR-803 treatment. CCK8 assays were performed after co-culture for 48 h. **C**, **D** Experimental scheme and migration ability of WT KYSE-30 and KYSE-450 cells with CM of CAFs with or without recombinant PLAU pretreatment in the presence or absence of IPR-803. The numbers of migrating cells were compared between the groups (the bottom panel). **E**–**H** Outline experimental scheme (**E**), tumor volume (**F**), and weights (**G**) and growth curves (H) of subcutaneous tumor xenografts established from co-implantation of WT KYSE-450 cells, and indicated MRC-5 cells in the presence or absence of IPR-803 treatment (female 6-week-old to 8-week-old nude mice, *n* = 6 per group). Three biological replicates were performed for in vitro assays. Three biological replicates were performed for in vitro assays. Data in bar charts are presented as the mean ± SD. *P < 0.05, **P < 0.01, ***P < 0.001, ****P < 0.0001 (Student’s *t*-test).

CAF heterogeneity is critical for promoting or inhibiting tumor, which is influenced by the paracrine system of other cells^4,18,30,31^. To further explore the effect of PLAU secreted by tumor cells on CAF heterogeneity, we detected the proliferation of ESCC cells cocultured with CAFs in the context of recombinant PLAU stimulation (Fig. 5A). CAFs subject to recombinant PLAU stimulation significantly promoted the proliferation of wild type (WT) KYSE-30 and KYSE-450 cells, and this effect was attenuated by the PLAU-uPAR inhibitor IPR-803 (Fig. 5B). After recombinant PLAU stimulation, conditional medium (CM) of CAFs also promoted migration of WT KYSE-30 and KYSE-450 cells, and IPR-803 eliminated this effect (Fig. 5C, D). Similarly, in vivo, subcutaneous tumor xenografts established from co-implantation of WT KYSE-450 and MRC-5 cells pretreated with TGFβ1 and PLAU grew faster than the controls without PLAU pretreatment, and this effect was also inhibited by IPR-803 (Fig. 5E–H).

### Tumor-secreted PLAU promoted the formation of inflammatory CAFs, which secreted IL8 via phosphorylation of Akt/NF-κB

**Fig. 6:**
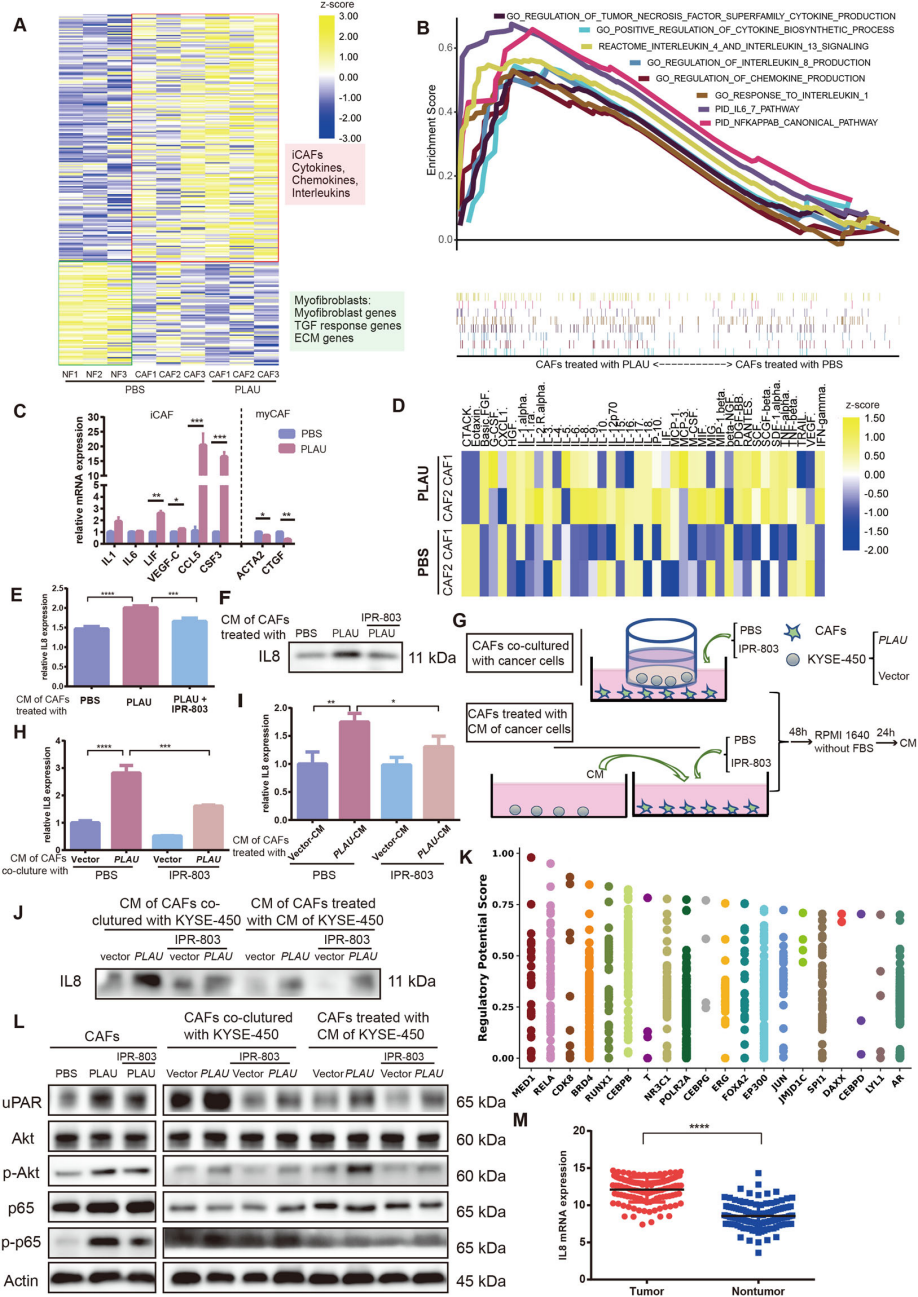
Tumor-secreted PLAU promoted the formation of inflammatory CAFs, which secreted IL8 via phosphorylation of Akt/NF-κB. **A** Transcriptional profile of NFs and CAFs with or without recombinant PLAU treatment. **B** Recombinant PLAU promoted activation of cytokine, chemokine, and interleukins pathways and NF-κB canonical pathways as demonstrated by GSEA. **C** RT-qPCR detection of markers of iCAFs and myCAFs in CAFs with or without recombinant PLAU treatment. **D** A 48-cytokine panel was used to characterize the CM of CAFs with or without recombinant PLAU treatment. **E**, **F** Detection of IL8 expression in CM of CAFs with or without recombinant PLAU treatment in the presence or absence of IPR-803, a uPA-uPAR inhibitor, by ELISA (**E**) and western blot (**F**). **G**–**J** Experimental scheme (**G**) and detection of IL8 expression in CM of indicated CAFs cocultured with *PLAU* overexpressing and vector KYSE-450 cells (**H**, **J**) or treated with CM of PLAU overexpressing and vector KYSE-450 cells (**I**, **J**), in the presence or absence of IPR-803 as assessed by ELISA (**H**–**I**) and western blot (**J**). The CM used for western blots was concentrated 40-fold. **K** Top 20 predicted transcription factors of CXCL1 predicted by the Cistome website. **L** Detection expression of uPAR, total and phosphorylated Akt and p65 of indicated CAFs. Three biological replicates were performed for in vitro assays. Data in bar charts are presented as the mean ± SD. *P < 0.05, **P < 0.01, ***P < 0.001, ****P < 0.0001 (Student’s *t*-test).

CAFs can be divided into inflammatory CAFs (iCAFs) with secretion phenotype and myofibroblasts (myCAFs) with contractility and matrix remodeling features^17,18^. To further explore the mechanism by which PLAU secreted by tumor cells promotes the tumor-promoting effect of CAFs, we performed RNA-seq of CAFs with or without recombinant uPA treatment and normal fibroblasts (NFs) obtained from the outgrowth of para-carcinoma tissues. CAFs interacts with tumor cells, and could promote progression of ESCC than NFs. iCAFs is prompting-tumor subtype, whereas myCAFs is inhibiting-tumor subtype^17^. Our results showed that compared with NFs, some gene sets of iCAFs, including of cytokines, chemokines, and interleukins, are highly expressed in CAFs, whereas some gene sets of myCAFs, including of myofibroblast genes, TGFβ-responsive genes, and extracellular matrix (ECM) genes, exhibit the opposite effects. Furthermore, after recombinant PLAU stimulation, CAFs exhibit upregulation of gene sets of iCAFs and downregulation of gene sets of myCAFs (Fig. 6A). In addition, PLAU stimulated the activation of cytokine or chemokine pathways in CAFs (Fig. 6B). We hypothesized that PLAU induce secretion of cytokines and chemokines from CAFs. PLAU promotes the formation of iCAFs. We detected the expression of iCAF and myCAF markers in CAFs with or without recombinant PLAU stimulation by RT-qPCR. The results obtained were consistent with the sequencing results, but PLAU treated CAFs lacked of IL6 RNA upregulation (Fig. 6C). Additionally, we collected the CM of CAFs with or without recombinant PLAU treatment to assess the expression of 48 cytokines or chemokines. The results showed that compared with the control group, CM of CAFs with PLAU stimulation exhibited increased expression of various cytokines, of which IL8 exhibit the most obvious expression (Fig. 6D).

Furthermore, western blot and ELISA were performed to further confirm that PLAU promotes CAF-mediated secretion of IL8, which can be reversed by IPR-803, a specific inhibitor of PLAU-uPAR (Fig. 6E, F). By assessing IL8 expression in CM of CAFs cocultured with tumor cells overexpressing *PLAU* and in CAFs stimulated by CM of tumor cells overexpressing *PLAU*, we found that PLAU secreted by tumor cells promoted CAFs secretion of IL8 and that PLAU-uPAR specific inhibitor IPR-803 reversed the promotion effect (Fig. 6G–J). In addition, enrichment analysis showed that the NF-κB pathway may be activated in CAFs with PLAU treatment (Fig. 6B), and NF-κB could transcriptionally regulate IL8 as predicted on the Cistrome website (Fig. 6K). We detected the expression of uPAR and phosphorylated Akt and p65 in CAFs stimulated by recombinant PLAU, CAFs cocultured with tumor cells overexpressing *PLAU*, and CAFs treated with CM of tumor cells overexpressing *PLAU*. The results showed that PLAU upregulates the expression of uPAR and phosphorylated Akt and p65 in CAFs, which can be reduced by IPR-803 (Fig. 6L). In addition, IL8 expression was increased in ESCC cancer tissues than in paracancer tissues (Fig. 6M), but expression levels were not related to the patient’s overall survival.

### IL8 secreted by CAFs upregulated PLAU expression in tumor cells

**Fig. 7:**
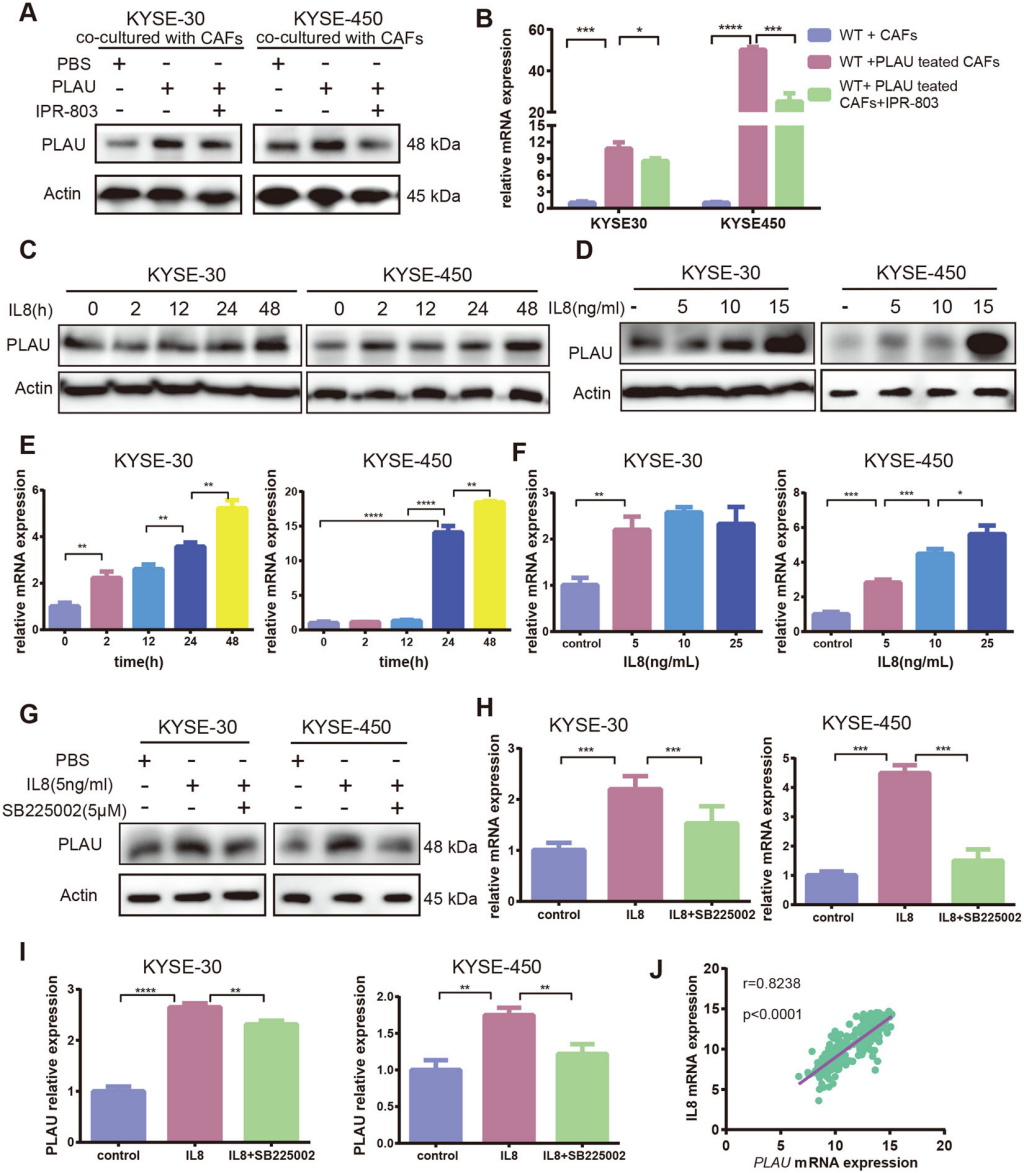
IL8 secreted by CAFs upregulated PLAU expression in tumor cells. **A**, **B** Detection of PLAU expression of WT KYSE-30 and KYSE-450 cells cocultured with CAFs treated with recombinant uPA in the presence or absence of IPR-803, a uPA-uPAR inhibitor, as assessed by western blot (**A**) and RT-qPCR (**B**). **C**–**F** Western blot and RT-qPCR were performed to detect PLAU expression of WT KYSE-30 and KYSE-450 cells exposed to recombinant IL8 treatments at different times (**C**, **E**) and concentrations (**D**, **F**). **G**–**I** Detection of PLAU expression in WT KYSE-30 and KYSE-450 cells with recombinant IL8 treatment in presence or absence of SB225005, a CXCR2 inhibitor, by western blot (**G**), RT-qPCR (**H**), and ELISA (**I**). **J** Correlation analysis of IL8 and *PLAU* expression based on GSE53625 data. Three biological replicates were performed for in vitro assays. Data in bar charts are presented as the mean ± SD. *P < 0.05, **P < 0.01, ***P < 0.001, ****P < 0.0001 (Student’s *t* test).

Numerous previous studies demonstrated that IL8 promotes ESCC proliferation and invasion^32–34^. We had confirmed that PLAU secreted by tumor cells promotes IL8 secretion by CAFs. We further explored and found that PLAU expression was increased in WT KYSE-30 and KYSE-450 cells cocultured with CAFs with uPA treatment. The PLAU-uPAR inhibitor IPR-803 attenuated this effect (Fig. 7A, B). In addition, we found that recombinant IL8 upregulates PLAU expression in WT ESCC cells in a time-dependent and concentration-dependent manner (Fig. 7C–F). Increased PLAU expression mediated by IL8 is reversed upon treatment with SB225005, a CXCR2 inhibitor (Fig. 7G, H). IL8 also upregulated PLAU secretion of tumor cells, which is also reversed by SB225005, a CXCR2 inhibitor (Fig. 7I). By analyzing GSE53625 data, we found that IL8 expression in ESCC tissue was positively correlated with *PLAU* expression (Fig. 7J).

## Discussion

**Fig. 8:**
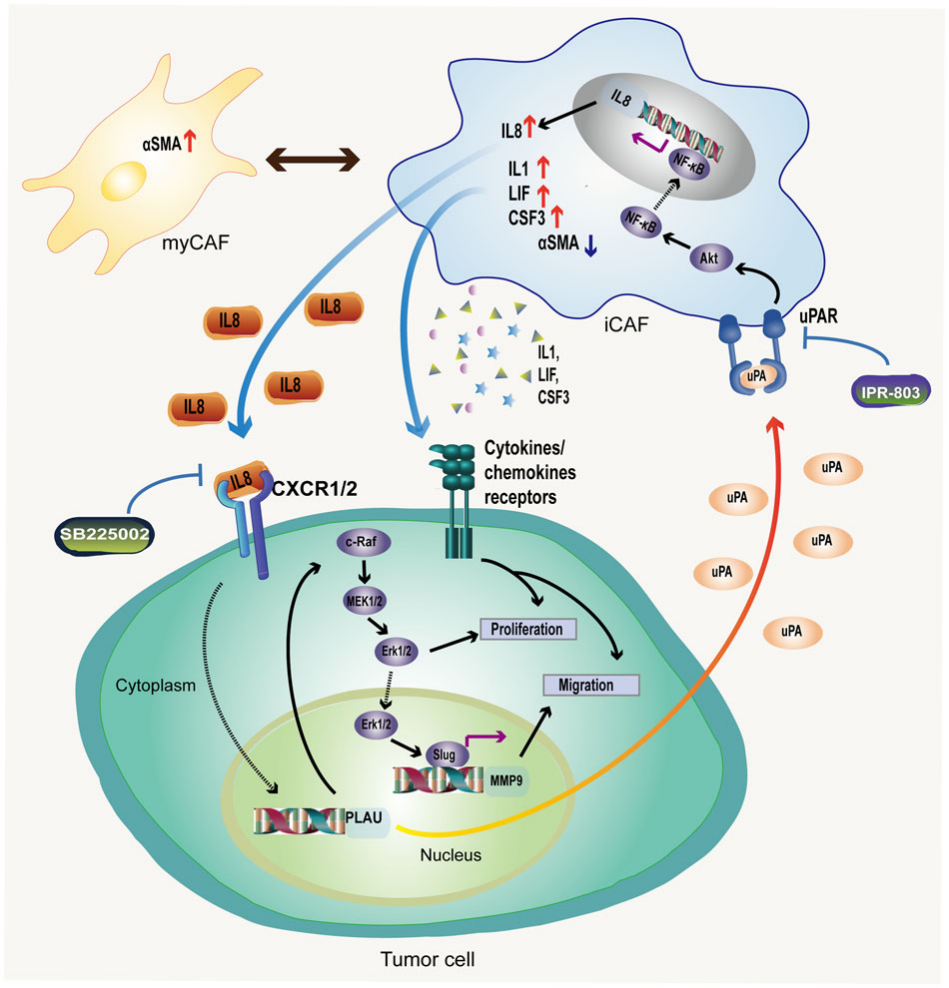
Research summary: PLAU promotes tumor cells proliferation and migration via MAPK/MEK/Erk/Slug/MMP9. Tumor cell secreted-PLAU promotes the conversion of fibroblasts to iCAFs that facilitate the expression and secretion of IL8 via uPAR/Akt/NF-κB, and this activity can be can be blocked by a PLAU-uPAR inhibitor (IPR-803). IL8-secreted by iCAFs upregulates PLAU expression.

In summary, PLAU promotes tumor cells proliferation and migration via MAPK/MEK/Erk/Slug/MMP9. Tumor cell secreted-PLAU promotes the conversion of fibroblasts to iCAFs that facilitate the expression and secretion of IL8 via uPAR/Akt/NF-κB, and this activity can be can be blocked by a PLAU-uPAR inhibitor (IPR-803). IL8-secreted by iCAFs upregulates PLAU expression (Fig. 8).

We found that overexpression of PLAU in ESCC is associated with poor prognosis, and *PLAU* potentially serves as a prognostic marker. Consistent with previous studies, high *PLAU* expression promotes the progression of ESCC^35^, and other various tumors, including breast, bladder, and lung cancer^21,22,27^. We found that *PLAU* promotes ESCC proliferation and tumor growth by activating the MAPK pathway. There is a close relationship between expression of PLAU and that of p38MAPK^36^. High *PLAU* mRNA level is a characteristic of cancer cells with functional TRAIL signaling^37^. PLAU plays a role in promoting metastasis in various tumors^27–29^. PLAU expression is increased in breast cancer CTC cells^28^. We found that *PLAU* promotes migration in ESCC, and upregulates the expression of Slug and MMP9 via the MAPK pathway. MMP9 plays an important role in ECM remodeling, which could promote tumor metastasis^38–40^. Consistent with our results, previous studies have also confirmed that PLAU regulates ECM remodeling of the extracellular matrix, affects cell adhesion, and promotes tumor metastasis^19^. Additionally, previous studies have showed uPAR combined with integrins active many intercellular signals in tumor cells^41^, and c-Raf could be downstream of integrins^42,43^. The link between uPAR/integrin and PLAU/c-Raf/MEK/Erk need furthermore research in the future.

CAFs are as heterogeneous as tumor cells^44^. Other cells influence CAF heterogeneity through paracrine methods. Different CAFs subgroups may secrete different cytokines to exert various functions, including promoting or inhibiting tumor progression^16–18^. In pancreatic cancers, IL1 secreted by tumor cells promotes the conversion of fibroblasts around tumor cells into iCAFs by activating the JAK/STAT pathway^18^. We confirmed that PLAU secreted by tumor cells promotes the tumor-promoting effect of CAFs through the transformation of inflammatory CAFs. PLAU increases the expression of gene sets of iCAFs, but decreases the expression of gene sets of myCAFs. PLAU promotes the activation of cytokine, chemokine, and interleukin signaling pathways in CAFs. PLAU not only promotes the increase iCAF marker expression but also the reduces the expression of myCAF markers. Assessment of the CM of CAFs subject to PLAU treatment reveals increased expression of most cytokines and chemokines. All findings indicate that PLAU promotes the transformation of fibroblasts to iCAFs, which plays a tumor-promoting effect. Furthermore, we also found after PLAU treatment, two of NFs also have upregulation of gene sets of iCAFs and downregulation of myCAFs (Fig S1). PLAU promotes NF-kB pathway activation in CAFs, which is an upstream transcription factor that regulates a variety of cytokines and chemokines^45^. iCAF has secretory properties. It not only promotes tumor progression and deterioration through paracrine methods but may also be related to the systemic response of tumors^18^.

We also found that tumor secreted-PLAU promotes CAF-mediated secretion of IL8 via phosphorylation of NF-κB. In addition, IL8 subsequently upregulates PLAU expression and secretion from tumor cells, which further promotes the progression of ESCC. IL8, which is also known as CXCL8, is a common chemokine that promotes neutrophil chemotaxis^46^. IL8 plays an important role in angiogenesis, survival, tumor stemness, invasion, metastasis, and immune cell infiltration in breast, lung, prostate, and pancreatic cancer^46^. In ESCC, IL8 directly binds to CXCR1/2 to promote invasion and migration^47,48^. IL8 also recruits myeloid-derived suppressor cells by chemotaxis to promote tumor cell growth^48^. IL8 is associated with progression, metastasis, inflammation, and poor prognosis in ESCC patients^47^. CAFs stimulated by PLAU upregulates IL8 expression and secretion via uPAR/Akt/NF-κB. In esophageal carcinoma, IL8 and IL1β are the main downstream effectors of NF-κB^40^. In conclusion, PLAU could promote progression of tumor cell, and tumor cell secreted-PLAU promotes the conversion of fibroblasts to iCAFs, a promoting-tumor type of CAFs.

## Supplementary information

Supplementary Tables

Supplementary figure or tables legends

Fig S1
