# Supplementary material for: PLAU directs conversion of fibroblasts to inflammatory cancer-associated fibroblasts, promoting esophageal squamous cell carcinoma progression via uPAR/Akt/NF-κB/IL8 pathway
Source: Cell Death Discov. 2021 Feb 11;7:32. doi: 10.1038/s41420-021-00410-6 (PMC7878926; doi:10.1038/s41420-021-00410-6)
Supplement: Supplementary file 1 — Supplementary Tables [file 41420_2021_410_MOESM1_ESM.docx]

**Supplementary Material**

**Table S1. Correlation analysis between the expression of PLAU and clinicopathological parameters in ESCC patients.**

| **Variables** | **All cases** | **PLAU** | | **χ**^2^ | ***P*^ab^** |
| --- | --- | --- | --- | --- | --- |
|  |  | **Low-expression** | **High-expression** |  |  |
| **Tissue types** |  |  |  |  |  |
| Marginal | 49 | 44 | 5 | 29.454 | <0.0001 |
| Tumor | 55 | 21 | 34 |  |  |
| **Lymph node metastasis**^c^ |  |  |  |  |  |
| yes | 28 | 12 | 16 | 1.268 | 0.260 |
| no | 25 | 7 | 18 |  |  |
| **Stage** |  |  |  |  |  |
| stage 0-2 | 23 | 14 | 9 | 5.259 | 0.022 |
| stage 3-4 | 25 | 7 | 18 |  |  |
| **Age** |  |  |  |  |  |
| ≤60years | 33 | 11 | 22 | 0.822 | 0.365 |
| >60years | 22 | 10 | 12 |  |  |
| **Gender** |  |  |  |  |  |
| Male | 37 | 16 | 21 | 1.227 | 0.268 |
| Female | 18 | 5 | 13 |  |  |
| **Differentiation** |  |  |  |  |  |
| Ⅰ | 10 | 6 | 4 | 2.465 | 0.116 |
| Ⅱ+Ⅲ | 45 | 15 | 30 |  |  |
| **Distant metastasis** |  |  |  |  |  |
| yes | 50 | 21 | 29 | 3.397 | 0.065 |
| no | 5 | 0 | 5 |  |  |

^a^ *P* values for comparing clinicopathological parameters in PLAU low-expression

group verus high-expression group.

^b^*P*<0.05 is considered significant.

^c^ missing 2cases

**Table S2. Patients’ characteristics from whom CAFs and NFs were obtained.**

|  | Age | Gender | Location | Differentiation | T_stage | N_stage | TNM | Distant metastasis |
| --- | --- | --- | --- | --- | --- | --- | --- | --- |
| Case1 | 64 | female | lower | II-III | T3 | N2 | IIIB | No |
| Case2 | 63 | female | Middle | III | T3 | N2 | IIIB | No |
| Case3 | 58 | male | lower | II | T3 | N1 | IIIA | No |

**Table S3. Primer sequences were used in our study**

| **Gene** | **Primer sequence** | |
| --- | --- | --- |
|  | **Forward** | **Reverse** |
| **β_Actin** | CTACCTCATGAAGATCCTCACCGA | TTCTCCTTAATGTCACGCACGATT |
| **PLAU** | CACACACTGCTTCATTGATTACCCA | TTTTCCACCTCAAACTTCATCTCCC |
| **IL1β** | GCCCAAGATGAAGACCAACCAGT | CCGTGAGTTTCCCAGAAGAAGAGG |
| **IL6** | CCTCCAGAACAGATTTGAGAGTAGT | CCTCCAGAACAGATTTGAGAGTAGT |
| **LIF** | TGAGGGCACTGGGGTTGAGGA | AGAAGGCCAAGCTGGTGGAGC |
| **VEGF-C** | AGAACAGGCCAACCTCAACTCAA | CTCCTTCCCCACATCTATACACACC |
| **CCL5** | CTGTTTCTGCTTGCTCTTGTCCT | TTTTGTAACTGCTGCTGTGTGG |
| **CSF3** | CCTGCATTTCTGAGTTTCATTCT | GCTGGGGAGCAGTCATAGTA |
| **ACTA2** | CCTTGAGAAGAGTTACGAGTTGC | ATGATGCTGTTGTAGGTGGTTT |
| **CTGF** | CCTGTCTTACTTTTCCGAAGGAC | CGTCAGGGCACTTGAACTCC |

**Table S4. Antibodies were used for western blot in our study**

| **Antibodies** | **Catalog no.** | **Company** |
| --- | --- | --- |
| **Anti-β_Actin-antibody** | A1978 | **Sigma Aldrich** |
| **Anti-PLAU-antibody** | ab169754 | **Abcam** |
| **Anti-c-Raf-antibody** | 53745 | **Cell Signaling Technology** |
| **Anti-p-c-Raf-antibody** | 9427 | **Cell Signaling Technology** |
| **Anti-MEK1/2-antibody** | A4868 | **ABclonal** |
| **Anti-p-MEK1/2-antibody** | 9154 | **Cell Signaling Technology** |
| **Anti-Erk1/2-antibody** | 4695 | **Cell Signaling Technology** |
| **Anti-p-Erk1/2-antibody** | 4370 | **Cell Signaling Technology** |
| **Anti-Slug-antibody** | 9585 | **Cell Signaling Technology** |
| **Anti-MMP9-antibody** | 13667 | **Cell Signaling Technology** |
| **Anti-IL8-antibody** | A2541 | **ABclonal** |
| **Anti-uPAR-antibody** | 12863 | **Cell Signaling Technology** |
| **Anti-Akt-antibody** | 4691 | **Cell Signaling Technology** |
| **Anti-p-Akt-antibody** | 4060 | **Cell Signaling Technology** |
| **Anti-p65(Ser536)-antibody** | 8242 | **Cell Signaling Technology** |
| **Anti-p-p65(Ser536)-antibody** | ab76302 | **Abcam** |
| **Anti-rabbit IgG, HRP-linked antibody** | 7074 | **Cell Signaling Technology** |
| **Anti-mouse IgG, HRP-linked antibody** | 7076 | **Cell Signaling Technology** |
