# Supplementary material for: PLAU directs conversion of fibroblasts to inflammatory cancer-associated fibroblasts, promoting esophageal squamous cell carcinoma progression via uPAR/Akt/NF-κB/IL8 pathway
Source: Cell Death Discov. 2021 Feb 11;7:32. doi: 10.1038/s41420-021-00410-6 (PMC7878926; doi:10.1038/s41420-021-00410-6)
Supplement: Supplementary file 2 — Supplementary figure or tables legends [file 41420_2021_410_MOESM2_ESM.docx]

**Supplementary Material Figure or tables legends**

**Table S1. Correlation analysis between the expression of PLAU and clinicopathological parameters in ESCC patients.**

**Table S2. Patients’ characteristics from whom CAFs and NFs were obtained.**

**Table S3. Primer sequences were used in our study**

**Table S4. Antibodies were used for western blot in our study**

**Figure S1. After PLAU treatment, two of NFs may have up-regulation of gene sets of iCAFs and down-regulation of gene sets of myCAFs.**
