# Supplementary figures and images for: PLAU directs conversion of fibroblasts to inflammatory cancer-associated fibroblasts, promoting esophageal squamous cell carcinoma progression via uPAR/Akt/NF-κB/IL8 pathway
Source: Cell Death Discov. 2021 Feb 11;7:32. doi: 10.1038/s41420-021-00410-6 (PMC7878926; doi:10.1038/s41420-021-00410-6)

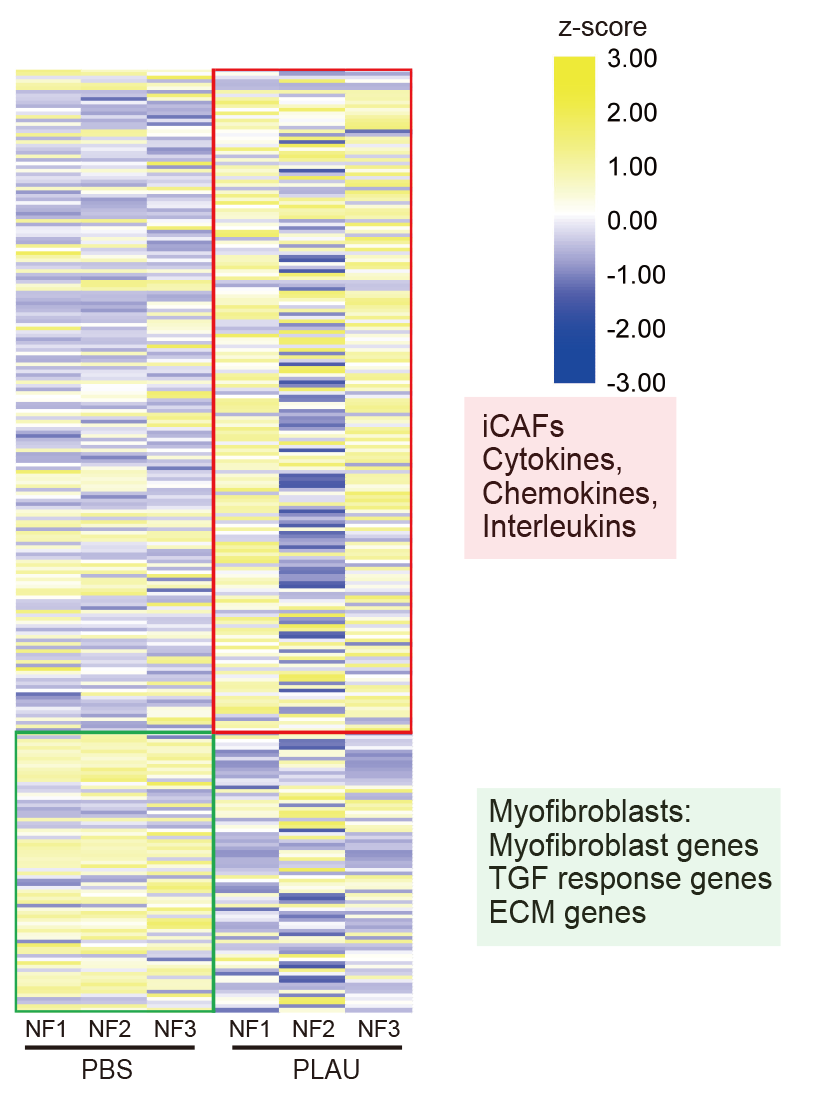

Supplement: Supplementary file 3 — Fig S1 [file 41420_2021_410_MOESM3_ESM.tif]
